# Characterization of Mediterranean Durum Wheat for Resistance to *Pyrenophora tritici-repentis*

**DOI:** 10.3390/genes13020336

**Published:** 2022-02-11

**Authors:** Marwa Laribi, Amor Hassine Yahyaoui, Wided Abdedayem, Hajer Kouki, Khaled Sassi, Sarrah Ben M’Barek

**Affiliations:** 1 LR14AGR01 Laboratory of Genetic and Cereal Breeding, National Agronomic Institute of Tunisia, University of Carthage, Tunis 1082, Tunisia; mar.wa199@hotmail.fr (M.L.); widedabdedayem@gmail.com (W.A.); khaledsassi1@gmail.com (K.S.); 2 CRP-Wheat Septoria Precision Phenotyping Platform, Tunis 1082, Tunisia; amor.yahyaoui@gmail.com (A.H.Y.); koukihajercm@gmail.com (H.K.); 3 International Maize and Wheat Improvement Center (CIMMYT), Carretera México-Veracruz, El Batan, Texcoco 56130, Mexico; 4 Regional Field Crops Research Center of Beja (CRRGC) BP 350, Béja 9000, Tunisia

**Keywords:** tan spot, durum wheat, landrace, phenotypic diversity, plant height, resistance, Mediterranean, Tunisia

## Abstract

Tan spot (TS), caused by the fugus *Pyrenophora tritici-repentis* (*Ptr*), has gained significant importance in the last few years, thereby representing a threat to wheat production in all major wheat-growing regions, including Tunisia. In this context, we evaluated a Mediterranean collection of 549 durum wheat accessions under field conditions for resistance to *Ptr* over two cropping seasons in Jendouba (Tunisia), a hot spot for *Ptr*. The relative disease severities showed significant phenotypic variation from resistance to susceptibility. The correlation between disease scores over the two trials was significant, as 50% of the accessions maintained good levels of resistance (resistant–moderately resistant). Seedling and adult-stage reactions were significantly correlated. The ANOVA analysis revealed that the genotype term is highly significant at the adult stage, thus emphasizing the high genetic variability of the tested accessions. Reaction-type comparison among and between countries revealed a high diversity of TS resistance. Plant height (PH) was negatively correlated to disease scores, indicating that PH might either have a significant effect on TS severity or that it can be a potential disease escape trait. The evaluation of this collection allowed for the identification of potential diverse resistance sources to *Ptr* that can be incorporated in breeding programs.

## 1. Introduction

The Mediterranean region is known as a major secondary center of durum wheat (*Triticum turgidum*), the domestication of which occurred in the region 12,000 years ago [1]. The genetic evolution of durum wheat in the Mediterranean region, as well as natural and human selection, led to the establishment of Landraces, with key quality traits including agronomic, quality characteristics, and adaptation to the region’s contrasting environment [2,3,4,5,6]. Durum wheat is still grown in the Mediterranean basin and North African countries (Algeria, Morocco, Tunisia, and Libya) mainly for its culinary final products, such as semolina, pasta, couscous, frike, and bourghul. North Africa produces 18.7 million tons (MT) of durum wheat, of which 1.5 MT is in Tunisia [7]. Since the 1970s, Tunisian farmers gradually abandoned landraces in favor of elite cultivars. Although, the currently cultivated cultivars are high yielding, they were found to be susceptible to multiple diseases, including tan spot, Septoria tritici blotch, and rusts [8,9,10,11]. Numerous studies evaluated Mediterranean durum wheat landraces for genetic diversity as well as resistance/tolerance to abiotic and biotic stresses. These studies revealed a high level of polymorphisms and allelic richness as well as a high adaptability to environmental conditions and fungal diseases [6,12,13,14]. Thus, the exploration of Mediterranean landraces could represent a possible source of novel genes or QTLs that can be introgressed into modern varieties.

Tan spot (TS) is a destructive foliar disease caused by *Pyrenophora tritici-repentis* (*Ptr*) that can affect durum and bread wheat, rye, barley, as well as numerous grass species [15,16,17,18]. This disease has been reported in all major wheat-growing regions, including North and South America, North and South Asia, Europe, and North Africa [19,20]. Tan spot is increasingly gaining importance in the Mediterranean region and has been reported in Algeria, Morocco, and Tunisia [11,21,22,23,24,25]. To date, *Ptr* is known to produce three well characterized Necrotrophic effectors (NEs)—one NE inducing necrosis termed Ptr ToxA and two NEs inducing chlorosis termed Ptr ToxB and Ptr ToxC [26,27,28]. Nevertheless, several recent studies reported the detection of additional NEs, referred to as Ptr ToxD [29,30,31,32,33]. A compatible reaction between these NEs and their corresponding host sensitivity genes, *Tsn1*, *Tsc2* and *Tsc1*, leads to infection [20,34,35]. The primary infection is caused by ascospores, while the secondary infection is caused by conidia leading to the establishment of tan-brown necrotic lesions surrounded by chlorotic halos, with small black points in the center of the lesions distinctive of tan spot [36,37]. Tan spot is known to cause up to 50% yield losses under favorable conditions [34,36]. For instance, moderately infected fields in Morocco induced 18% yield losses [38]. In Tunisia, tan spot disease is an emerging threat to wheat production [11,14]. Although no studies were carried out to assess the extent of yield losses, recent studies about *Ptr* in Tunisia revealed a high morphological, phenotypic, and genotypic diversity [19,24,25,39]. Six races, namely, 2, 4, 5, 6, 7, and 8, were identified in addition to atypical isolates that were able to cause necrosis on the differential line ‘Glenlea’ but lacked the expected *ToxA* gene [19,24,25]. The homothallic nature of the pathogen, its virulence and the wide adoption of conservation practices, as well as the wide cultivation of susceptible cultivars, contributed to the high genetic diversity of the pathogen [11,25].

During the last three years of surveys, the most cultivated cultivars in Tunisia were found to be susceptible to tan spot disease in all wheat-growing regions [11]. Fungicides, cultivar mixtures, crop rotation, and host resistance are considered to be the most effective sustainable strategies for tan spot disease management [16,35,40,41]. In this context, this study aims to evaluate a collection of Mediterranean durum wheat accessions originating from Algeria, France, Italy, Portugal- and Spain for resistance to *P. tritici-repentis* under field conditions at the seedling and adult growth stages over two cropping seasons. The same collection was subsequently compared for resistance to tan spot and Septoria tritici blotch diseases [41,42]. The association of disease development to plant height (PH) was also investigated in relation to tan spot development.

## 2. Materials and Methods

### 2.1. Plant Materials

A collection of 549 Mediterranean, mainly durum wheat accessions, provided by USDA-NSGC (Aberdeen, ID, USA) were evaluated for two years (2018–2019 and 2020–2021) for resistance to *P. tritici-repentis* at the adult growth stage under field conditions at the CRP Wheat Septoria Precision Phenotyping Platform, experimental station located at Kodia (36°32′51.89 N, 9°0′40.73 E)-National Institute of Field Crops (Bou Salem, Tunisia), a hot spot for *Ptr* (Figure S1). Subsequently, all accessions were evaluated for resistance to *Ptr* at the seedling growth stage under field conditions during the 2018–2019 cropping season. The collection comprised 540 durum wheat accessions and only nine bread wheat accessions and included 360 landraces, 44 cultivars, 13 genetic materials, 62 breeding material, and 70 accessions of unknown improvement status from five different countries. The accessions originated from Algeria (147 accessions), France (12 accessions), Italy (126 accessions), Portugal (245), and Spain (19 accessions) (Figure S1, Table S1) [11,42]. All accessions were planted on 13 November 2018 and 16 November 2020, in a wheat after wheat monoculture system.

### 2.2. Experimental Design and Inoculation

An augmented experimental design with unreplicated entries but replicated checks was implemented during both year trials. Plots consisted of two rows of 1 m length. Spacings between plots and blocks were 0.5 and 1 m, respectively. Four local checks were used in each block, with a total of 36 checks. The checks include varieties known to be moderate (‘INRAT 100’), susceptible (‘Karim’ and ‘Nasr’), and resistant (‘Salim’) to tan spot disease. The susceptible variety ‘Nasr’ was also planted in the middle of the block and served as disease spreader to induce infection and ensure optimal disease development and distribution among and within plots. To further induce infection, infested straw from the previous cropping seasons was incorporated into the soil with a rotary harrow. Additional straw inoculations were performed by evenly spreading freshly cut infected wheat straw over the experimental plots and disease spreader with an inoculum density of approximately 500 g·m^−2^ at GS10 [43]. Irrigation was also applied to ensure favorable conditions for TS development and standard wheat agronomic practices were carried out. The average of the replicated checks was used to verify the uniformity of infection and to classify the accessions tested in this study based on their levels of resistance/susceptibility (Table S2). Plant height (PH) was measured using a yardstick (in cm), at maturity, from ground level to the tip of the spike (including awns) at both 2018–2019 and 2020–2021 seasons.

### 2.3. Climatic Conditions

Weather conditions including precipitations, temperature (T) (minimum, mean, and maximum), and relative humidity (RH) were recorded over the two cropping seasons from November to May (2018–2019 and 2020–2021) [44]. Data are illustrated in Figure S2.

### 2.4. Confirmation of the Pathogen

Samples were collected from debris and leaves at different growth stages for pathogen morphological confirmation. Leaves with typical tan spot symptoms were subsequently cut into small pieces and placed in petri dishes, each containing two layers of sterile filter paper moistened with sterile distilled water. These plates were incubated under fluorescent light for 24 h at 20 °C and then transferred to darkness at 15 °C for 18 to 24 h to promote conidial production. Leaf fragments were then examined using 40× binocular magnifiers to observe the presence or absence of conidia and conidiophores of *Ptr*. Single conidia identified as *Ptr*, were transferred to V8-PDA medium (150 mL of V8 juice, 10 g of Potato Dextrose Agar, 3 g of CaCO_3_, 10 g of water agar, and 850 mL of distilled water) and incubated at 20 °C. Debris were also examined under microscope to confirm the pathogen identity.

### 2.5. Tan Spot Evaluation

All accessions were evaluated for tan spot resistance/susceptibility under field conditions. The inoculated plants were evaluated for the severity of their reaction to *P. tritici-repentis* infection at GS13–GS20 [43] using a slightly modified 0–5 lesion rating scale [14]. Briefly, scores equal to 5 indicated susceptibility, while those equal to 4 indicated a moderately susceptible reaction of the genotype. Scores equal to 3 indicated a moderately resistant reaction of the genotype, while those equal or less than 2 indicated resistance. The comparison between seedling and adult growth stages resistance was crucial, as resistance expression may differ according to the plant development stage. Hence, all accessions were assessed for disease resistance under field conditions at three-four consecutive time points at the adult stage (GS55) [43] with a 7 day interval between each evaluation, over the two-year trials. These multiple observations allowed for the calculations of the area under the disease progress curve (AUDPC) and the relative area under the disease progress curve (rAUDPC). The disease progression was estimated by measuring the incidence and severity based on the double-digit scale (00–99) [45], where the first digit indicates disease incidence on the infected plants, and the second digit refers to the severity of infection. The AUDPC and rAUDPC were calculated according to the formulas shown below [46] and allowed for quantitative analyses of the temporal differences in disease progress:

(1) $$\mathrm{AUDPC}=\overset{n-1}{\underset{i=1}{\sum }}\frac{y_{i}+y_{i+1}}{2}\times \left(t_{i+1}-t_{i}\right)$$

where:

y_i_ = disease severity at time t_i_;

t_(i+1)_ − t_i_ = time interval (days) between two disease scores;

n = numbers of scoring events.

(2) $$\mathrm{rAUDPC}=\frac{\mathrm{AUDPC}\;\left(\mathrm{genotype}\right)}{\mathrm{AUDPC}\;\left(\mathrm{Nasr}\right)}$$

where Nasr is the susceptible check of the corresponding trial.

### 2.6. Statistical Analysis

R software version 4.1.2 (R Foundation for Statistical Computing (R Core Team (2021)) [47] was used for all data analysis. Principal Component Analysis (PCA) was performed using the R package ‘MASS’ [48]. The determination and visualization of clusters was performed using R packages ‘factoextra’, ‘cluster’, and ‘stats’ [49,50]. The coefficient of correlation between variables (seedling and adult reaction, and PH) was determined with ‘cor.test’ function from the R package ‘stats’, and the analysis of variance (ANOVA) was performed with ‘aov’ function from the R package ‘stats’.

## 3. Results

### 3.1. Reactions of Genotypes across the Two Trials

As a part of the evaluation process, infected wheat leaves and debris were collected and analyzed morphologically to confirm the presence of *Ptr*. The identification of typical conidia of *Ptr* from collected leaf samples confirmed the infection with tan spot (Figure S3), and several obtained isolates have been published in Laribi et al. [24,25]. In addition, the collected debris contained spherical black pseudothecia visible to naked eyes. The latter were smashed and further examined under a microscope for the identification of asci and ascospores of *Ptr* (Figure S3).

The rAUDPC scores of the resistant and susceptible checks (‘Salim’ and ‘Nasr’) implemented in the experimental design were used to classify the Mediterranean wheat accessions for TS resistance under field conditions (Table 1 and Table S1).

**Figure 1:**
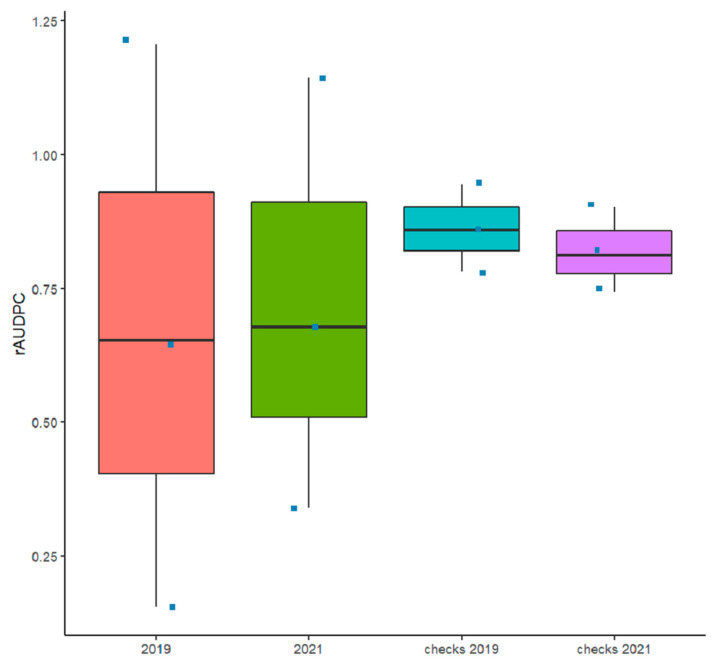
Box plots of the relative area under the disease progress curve (rAUDPC) of all the checks and accessions inoculated with *Pyrenophora tritici-repentis* under field conditions in 2018–2019 and 2020–2021 cropping seasons.

The comparison of the mean rAUDPC and standard deviation of the tested germplasm as well as the checks used in this study showed highly consistent overall disease pressure (Figure 1 and Table S2). The environmental conditions (temperature range, precipitations, and relative humidity (Figure S2)) coupled with the inoculation methodology used in this study were favorable for disease development among and within plots and blocks during both cropping seasons. The overall disease pressure for all tested accessions was similar between the two cropping seasons, similar to the disease pressure for checks (Figure 1).

**Figure 2:**
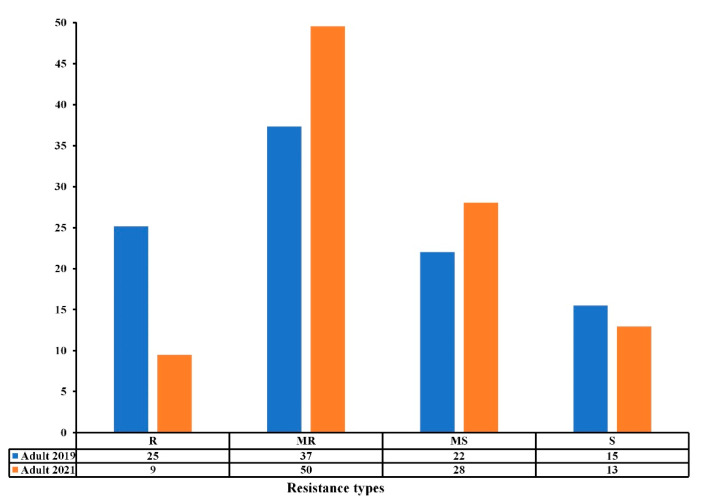
Resistance levels (%) of the Mediterranean wheat accessions to tan spot disease at adult stage in field conditions in the 2018–2019 and 2020–2021 cropping seasons. The resistance types are classified as resistant (R), moderately resistant (MR), moderately susceptible (MS), and susceptible (S).

All accessions responded differentially to TS under field conditions at the adult growth stage over the two-year trials, exhibiting reactions that ranged from susceptible (S) to resistant (R) (Table 1 and Table S1) (Figure 2). Our results show that 25% were resistant (R), 37% moderately resistant (MR), 22% moderately susceptible (MS), and 15% susceptible (S) in the 2018–2019 cropping season, while 9%, 50%, 28% and 13% were R, MR, MS, and S in the 2020–2021 cropping season. The number of susceptible accessions remained approximately the same from 2018–2019 to 2020–2021, while the number of resistant accessions decreased towards MR and MS reactions (Figure 2). The Pearson correlation coefficient (*r*) between the experiments at the adult stage was highly significant (*r* = 0.531, *p* ≤ 0.001) (Table 2), where 50% of the accessions maintained good levels of resistance (R-MR) across the two trials (Figure 2, Table S1).

The analysis of variance showed that the genotype is highly significant (*p* ≤ 0.001), indicating that the observed variation in the disease response is mainly due to the level of genetic variation between the accessions (Table 3). A moderately significant difference in disease response means between years (2019 and 2021) was revealed by ANOVA analysis. Moreover, no significant genotype × year interaction was observed (Table 3).

### 3.2. Comparison of Seedling and Adult Plant Resistance

**Figure 3:**
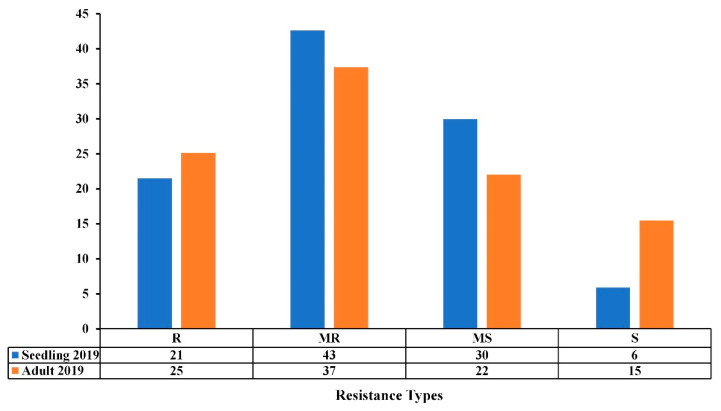
Resistance levels (%) of the Mediterranean wheat accessions to tan spot disease at seedling and adult stages under field conditions in the 2018–2019 cropping season. The resistance types are classified as resistant (R), moderately resistant (MR), moderately susceptible (MS), and susceptible (S).

All accessions were evaluated for seedling and adult resistance/susceptibility under field conditions during the 2018–2019 cropping season. On average, 64% and 62% of the accessions had good levels of resistance (R-MR) at both the seedling and adult stages, while 36% and 38% showed susceptible reactions (MS-S) to *Ptr* (Figure 3). The Pearson correlation coefficient (*r*) between the seedling and adult stages was highly significant (*r* = 0.287, *p* ≤ 0.001) (Table 2). The analysis of variance showed that the seedling reaction is highly significant (*p* ≤ 0.001), thereby indicating that the observed disease response at the adult stage is highly associated to that of seedling stage (Table 3). The same level of 69% of R-MR accessions at the seedling stage remained as R-MR at the adult stage during the 2018–2019 cropping season (Table S1).

### 3.3. Geographical Distribution of the Resistant and Susceptible Accessions

**Figure 4:**
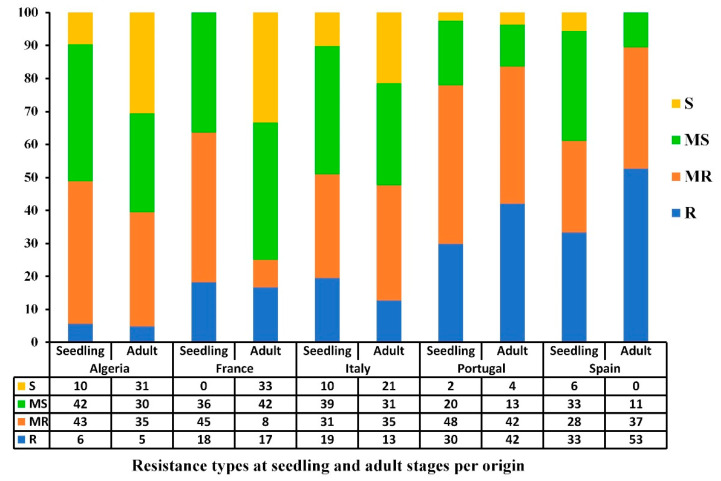
Bar graph showing the resistance levels (%) of the Mediterranean wheat accessions against *Pyrenophora tritici-repentis* at seedling and adult growth stages based on the country of origin under field conditions during the 2018–2019 cropping season. The resistance types are classified as resistant (R), moderately resistant (MR), moderately susceptible (MS), and susceptible (S).

The resistance levels varied between the stages of development and countries of origin (Figure 4). At the seedling stage, over 50% of accessions from all five Mediterranean countries (Algeria, France, Italy, Portugal, and Spain) had good resistance levels (R-MR) at the seedling stage, with Portugal having the highest percentage of resistant accessions (78%), followed by France and Spain (63% and 61%, respectively). At the adult stage, Spain and Portugal had the highest levels of resistance, where 90% and 84% of the accessions were R and MR. In contrast, France had the lowest frequency of resistant accessions (25% R-MR). The Algerian, Italian, and Portuguese populations had a slight to-non-existent change in resistance level between the seedling and adult stages, whereas the French and Spanish populations were highly variable (Figure 4). Although a highly significant correlation was observed between seedling and adult stages reactions for the 2018–2019 cropping season (Table 2), some variability between countries was observed; for instance, accessions from France and Spain showed a variability in their disease response from seedling to adult stage while accessions from Algeria, Italy, and Portugal maintained similar levels of resistance.

**Figure 5:**
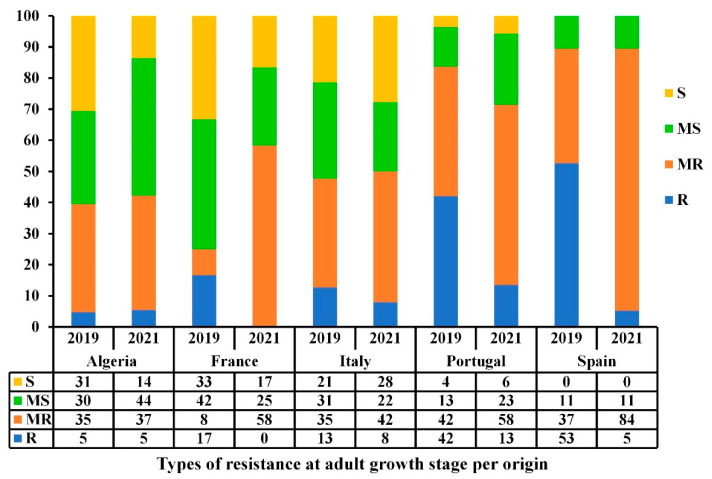
Bar graph showing the resistance levels (%) of the Mediterranean accessions against *Pyrenophora tritici-repentis* at adult growth stage based on the country of origin under field conditions during the 2018–2019 and 2020–2021 cropping seasons. The resistance types are classified as resistant (R), moderately resistant (MR), moderately susceptible (MS), and susceptible (S).

A high diversity within and between countries of origin was observed when comparing the resistance levels over the two-year trials (Figure 5). ANOVA analysis showed that the origin of accessions is highly significant (*p* ≤ 0.001) at the adult stage over the two testing years, thereby indicating that the observed disease response at the adult stage is highly associated to the geographical origin of accessions (Table 3). The same trend was observed for accessions from Algeria and Italy that showed low variability in terms of frequencies of resistance (R-MR)/susceptibility (MS-S) levels among and between years (Figure 5). Accessions from France, Portugal, and Spain showed a variability in the frequency of resistant and susceptible reactions between years. Although populations from the latter regions had good levels of resistance, there was a shift in the frequencies of disease reactions from R to MR between years. Portugal and Spain had the lowest frequencies of MS-S accessions over the two years. Although a highly significant correlation was observed between adult-stage reactions over the two years (Table 2), a variability between countries of origin, particularly for accessions from France and Spain was noticed (Figure 5).

### 3.4. Resistance Levels to Tan Spot with Regard to the Level of Improvement

When comparing the frequency of resistance (R-MR) of all accessions based on their level of improvement at both the seedling and adult stages, a high diversity was observed with regard to TS resistance. At the seedling stage, the percentage of R-MR accessions varied depending on the level of improvement from 30% for cultivars to 73% for accessions with unknown improvement status (Table 4). At the adult stage, the percentage of R-MR accessions varied from 18 to 85% over two years of trials (Table 4). Genetic material and Landraces had the highest levels of resistance with 85% and 49%, respectively, while cultivars had the lowest level of R-MR with 18% (Table 4). The latter results further confirmed the ANOVA analysis that showed that the Level of improvement of accessions is highly significant at the adult stage over two years (*p* ≤ 0.001) (Table 3). The percentage of R-MR accessions remained similar from 2019 to 2021 for all levels of improvement further confirming the significant correlation between adult responses over the two years of trials (Table 2).

### 3.5. Association between Disease and Plant Height

**Figure 6:**
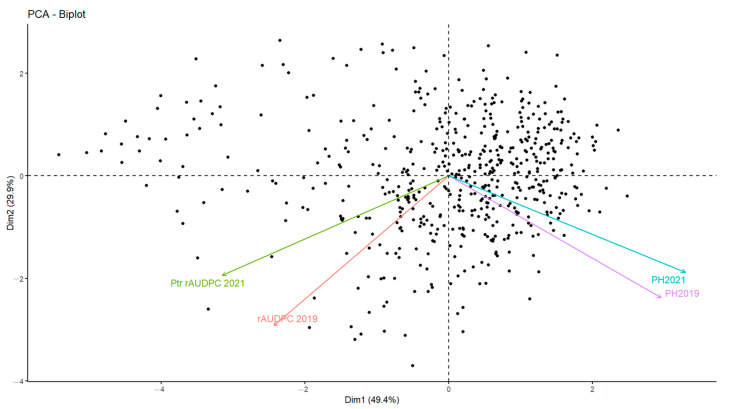
Principal component analysis (PCA) representing the contribution of two factors: plant height (PH), and rAUDPC scores for the 549 Mediterranean wheat accessions tested in this study over two years of trials.

In addition to the disease response, plant height (PH) was recorded to identify any association to tan spot infection. A significant variation within the collection of accessions in relation to this trait was observed (Table S1). PH ranged from 60 to 195 cm for 2019 and from 75 to 190 cm for 2021. To further investigate the effect of PH on tan spot infection, a principal component analysis (PCA) was conducted using PH and rAUDPC as parameters. The results show two dimensions of PCA, explaining 79.3% of data variance (Figure 6). The first dimension accounted for 49.4% of the variances, while the second dimension accounted for 29.9% of variances.

Significant correlation (*r* = 0.605, *p* ≤ 0.001) was detected between PH values measured in 2019 and 2021 (Table 2). A negative, moderately significant correlation between PH and rAUDPC values was found (*r* = −0.086, *p* ≤ 0.05) for 2019, while for 2021 a negative, highly significant correlation between PH and rAUDPC values was noticed (*r* = −0.347, *p* ≤ 0.001). ANOVA analysis revealed the significant negative effect of PH (*p* ≤ 0.001) on adult disease reactions (Table 2).

**Figure 7:**
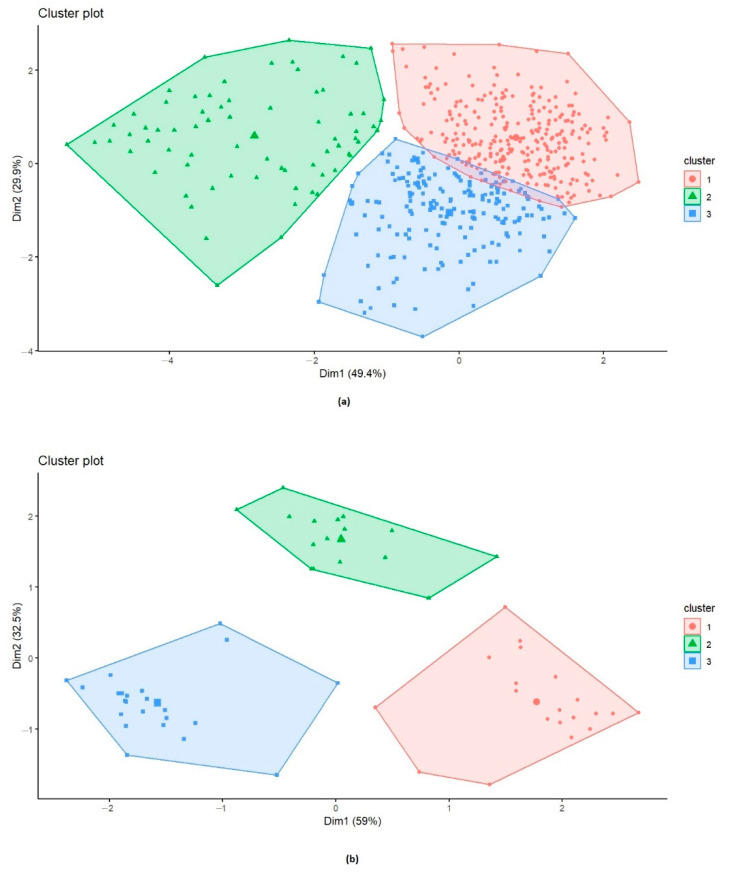
(**a**) Clustering of 549 wheat accessions representing the contribution of plant height (PH) and the relative area under disease progression curve (rAUDPC) over two years of trials (2019/2021); Cluster 1, 2, and 3 included 287, 71, and 191 accessions, respectively. (**b**) Clustering of 59 wheat accessions representing the contribution of plant height (PH) and the relative area under disease progression curve (rAUDPC) over two years of trials (2019/2021); Cluster 1, 2, and 3 included 20, 16, and 23 accessions, respectively.

To classify the accessions, a cluster analysis was conducted and revealed the presence of three clusters (Figure 7a). The first cluster (red) included most of the accessions (287 accessions). Cluster 3 (blue) was the second largest cluster, with 191 accessions, while Cluster 2 (green) contained 71 accessions. PH ranged from 95 to 185 cm for accessions of Cluster 1, from 60 to 160 cm for Cluster 2, and from 100 to 195 cm for Cluster 3. Cluster 1 included mostly R-MR accessions across the two years of trials (277 and 243 accessions in 2019 and 2021, respectively) and no susceptible accessions. Accessions in Cluster 2 were mostly MS-S (48 and 64 accessions in 2019 and 2021, respectively). Accessions in Cluster 3 were mostly MR-MS (128 and 162 accessions in 2019 and 2021, respectively) (Table S1).

A second cluster analysis was conducted with accessions that maintained their R or S reactions over the two years of trials, in order to differentiate between them. Accessions with MR and MS reactions were eliminated from this analysis. In total, 59 accessions were clustered into three different clusters (Figure 7b). The results show two dimensions explaining 91.5% of data variance. The first dimension accounted for 59% of the variances while the second dimension accounted for 32.5% of the variances. Cluster 1 (red) comprised 20 accessions, Cluster 2 (green) contained 16 accessions, while Cluster 3 (blue) comprised 23 accessions. Cluster 3 comprised 93.5% R accessions and 6.5 % S accessions with PH ranging from 105 to 180 cm. Cluster 1 included 92.5% of S accessions with PH ranging from 60 to 135 cm, and Cluster 2 comprised only S accessions with PH ranging from 110 to 195.

### 3.6. Distribution of the Reaction Types among and between Populations

**Figure 8:**
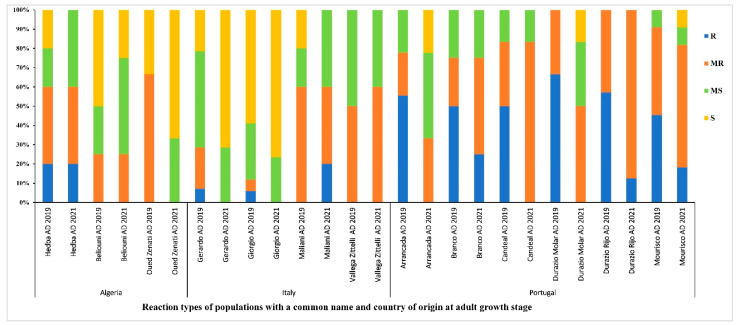
Disease reaction at adult growth stage of populations with a common name and country of origin, under field conditions during the 2018–2019 and 2020–2021 cropping seasons. AD refers to adult-stage reaction. R, MR, MS, and S refer to resistant, moderately resistant, moderately susceptible and susceptible reactions, respectively.

Accessions with a common name but different PI/Cltr numbers (USDA reference identifier) from the same or different countries of origin were compared for tan spot disease resistance/susceptibility at the adult stage during the 2018–2019 and 2020–2021 cropping seasons. Populations from Portugal had the highest level of resistance (R-MR) (Figure 8). Populations from Algeria and Italy had lower levels of resistance compared to populations from Portugal. Populations ‘Beliouni’ from Algeria, ‘Giorgi and Maliani’ from Italy, and ‘Arrancada’, ‘Branco’, ‘Candeal’, ‘Durazio Molar’, ‘Durazio Rijo’, and ‘Mourisco’ from Portugal maintained a good level of resistance (above 50% R-MR). Populations such as ‘Oued Zenati’ from Algeria and ‘Gerardo’ from Italy showed low levels of resistance in both cropping seasons (Figure 8). The ‘Arrancada’ population from Portugal and ‘Maliani’ from Italy showed a low variability in resistance between years, while other populations, such as ‘Candeal’ from Portugal, showed an important resistance/susceptibility variation between the trials (Figure 8).

**Figure 9:**
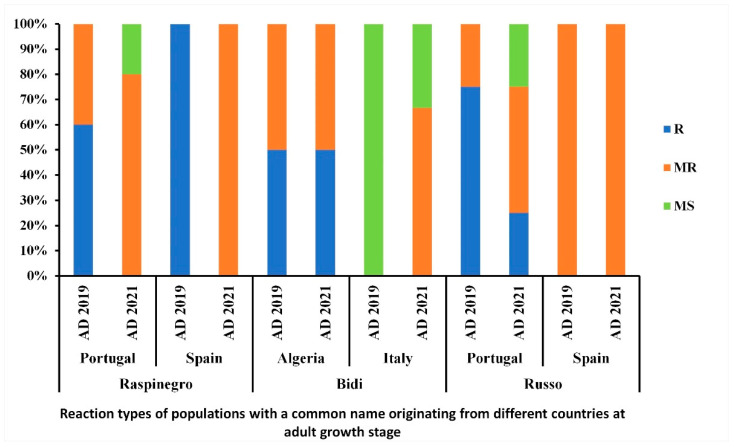
Disease reaction at adult growth stage of populations with a common name originating from different countries, under field conditions during the 2018–2019 and 2020–2021 cropping seasons. AD refers to adult-stage reaction. R, MR, and MS refer to resistant, moderately resistant and moderately susceptible reactions, respectively.

Levels of resistance/susceptibility of populations with the same common name but originating from different regions were also compared in this study (Figure 9). The population ‘Raspinegro’ was scored R-MR independently from the year-trial or origin of accessions. Meanwhile, the populations ‘Bidi’ and ‘Russo’ displayed a variable level of resistance and susceptibility with regard to the region of origin of the accessions (Figure 9).

### 3.7. Multiple Disease Resistance: TS and STB Association with Regard to Plant Resistance

**Figure 10:**
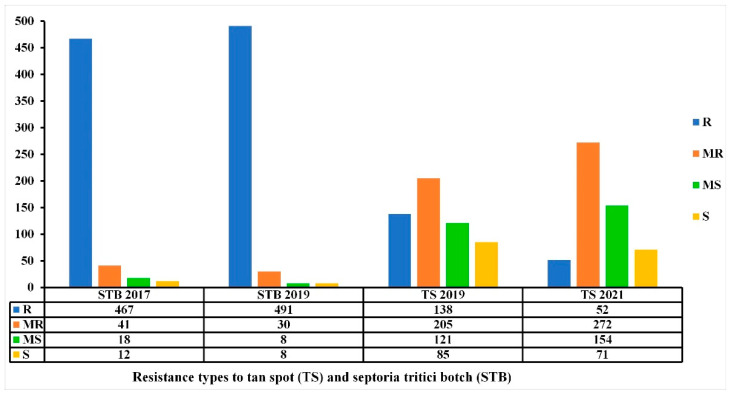
Resistance levels (number of accessions) of the 538 Mediterranean wheat accessions to tan spot (TS) and Septoria tritici blotch (STB) diseases at adult stage in the field conditions at kodia experimental station in the 2017–2018 and 2018–2019 cropping seasons for STB and 2018–2019 and 2020–2021 cropping seasons for TS.

Among the 549 Mediterranean wheat accessions tested in this study for TS resistance, 538 were also tested for STB resistance during two copping seasons (2016–2017 and 2018–2019) at the same location [42]. The Pearson correlation coefficient was highly significant (*r* = 0.289, *p* ≤ 0.001 and *r* = 0.328, *p* ≤ 0.001) for the overall STB-TS adult and STB-TS seedling, respectively. The overall results showed that these accessions were more susceptible to *Ptr* infection (Figure 10, Table S1).

The comparison of STB and TS data showed that among the 447 accessions that remained R-MR for STB disease over the two-year trials, 222 accessions (49.66%) remained R-MR for TS over the two trials (Figure 10, Table S1). The STB resistance levels between 2016/2017 and 2018/2019 were not variable, contrary to the TS resistance levels 2018/2019 and 2020/2021, where the number of R accessions decreased and the number of MR-MS accessions increased (Figure 10).

**Figure 11:**
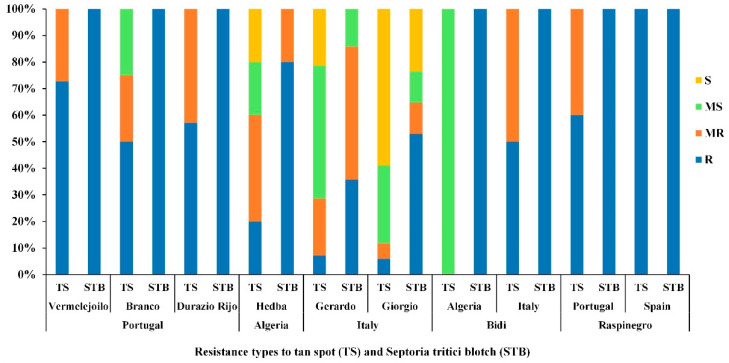
Disease reaction for tan spot (TS) and Septoria tritici blotch (STB), at adult growth stage of populations with a common country of origin (Portugal, Algeria and Italy) and populations with a common name originating from different countries (‘Bidi’ and ‘Raspinegro’), under field conditions during the 2018–2019 cropping season. R, MR, MS and S refer to resistant, moderately resistant, moderately susceptible and susceptible reactions, respectively.

Accessions with a common name but different PI/Cltr numbers (USDA reference identifier) from the same or different countries of origin were compared for TS and STB diseases resistance/susceptibility at the adult stage during the 2018–2019 cropping season [42] (Figure 11).

Populations (‘Vermelejoilo’, and ‘DurazioRijo’) from Portugal, (‘Bidi’) from Italy, and (‘Raspinegro’) from both Spain and Portugal had the highest level of resistance (R-MR) for both TS and STB diseases (Figure 11). The Italian populations (‘Gerardo’ and ‘Giorgio’) and Algerian populations (‘Hedba’ and ‘Bidi’) showed different levels of resistance/susceptibility for TS and STB and were more susceptible than the other populations (Figure 11). Levels of resistance/susceptibility of populations with the same common name (‘Bidi’ and ‘Raspinegro’) but originating from different regions were also compared (Figure 11). Population ‘Raspinegro’ was R-MR to both TS and STB independent of their origin. Meanwhile, population ‘Bidi’ displayed a variable level of resistance and susceptibility between the country of origin (Algeria, Italy) (Figure 11).

## 4. Discussion

Tan spot is among the rapidly emerging diseases threatening wheat production in Tunisia and can incur important yield losses under favorable conditions for disease development. Recent studies on *Pyrenophora tritici-repentis* in Tunisia revealed high phenotypic and genotypic diversity of *Ptr* in Tunisia. Hence, phenotyping wheat populations from the Mediterranean region under favorable conditions can lead to the discrimination between resistant and susceptible accessions [14,19,24,25]. In this study, 549 wheat accessions that comprised mainly landraces from five Mediterranean countries were assessed for resistance/susceptibility to TS disease in Tunisia over two cropping seasons at the adult stage. In addition, in the 2018–2019 cropping season, these accessions were also evaluated at the seedling stage. Furthermore, the climatic conditions in the Jendouba region (Figure S2) along with the artificial inoculation with infested straws and irrigation allowed for a uniform and optimal infection distribution. A variability in reactions to TS was observed between genotypes at both seedling and adult stages; several phenotypic classes were established (R, MR, MS, and S) and novel sources of resistance to TS were identified. The comparison of seedling and adult resistance revealed a highly significant correlation, similar to a recent study by Laribi et al. [14] under the same conditions, and to other studies [51,52,53,54]. During the testing period (2018–2019), 69% of accessions remained R-MR at both growth stages; therefore, screening for tan spot disease resistance at the seedling stage can allow for the elimination of most susceptible materials, avoiding costly and lengthy confirmations of field resistance as well as allowing one to reduce the number of accessions to be further tested in replicated field trials. This significant correlation may suggest the presence of all-stage resistance sources, QTLs or genes that are present, and most likely sustainable, at all plant growth stages [55]. Although the results of seedling–adult-stage responses to TS were highly correlated, we opted to focus on adult-stage resistance on the following year trial (2020–2021), as screening accessions for both stages under field conditions is labor intensive and time consuming, besides the fact that adult-stage resistance is preferred by breeders over seedling resistance as it represents a non-race-specific resistance to TS, which is a more sustainable form of resistance [34,35]. The accessions that exhibited different reactions between seedling and adult reactions, R-MR at the adult stage but MS-S at the seedling stage, could harbor a form of adult plant resistance (APR) that confers incomplete or partial resistance in the field [56]. The ANOVA analysis revealed that the genotype was highly significant at the adult stage, which indicates the diverse genetic background of the accessions tested in this study. Accessions that were R-MR in 2018–2019 but shifted to MR in 2020–2021 could carry combinations of major/minor resistance genes. Thus, a genome-wide association mapping study of these accessions along with the accessions that maintained an R-MR reaction over the two years of trials may lead to the identification of novel APR genes/QTLs that can be effective regardless of the environmental conditions and the plant growth stage. These accessions can be integrated in breeding for tan spot resistance in areas where the fungal population is highly diverse, such as Tunisia, where races 2, 4, 5, 6, 7, and 8 were identified in addition to the recent detection of atypical isolates that were able to cause necrosis on the differential line ‘Glenlea’ but lacked the expected *ToxA* gene [14,19,24,25]. As the breakdown of resistance could occur over all levels of resistance types and growth stages, it would be crucial to pyramid minor and major genes in order to avoid such an event, especially when breeding for resistance for a fungal disease that is highly diverse. The number of resistant accessions decreased towards MR and MS reactions from 2018/2019 to 2020/2021. The precipitations, relative humidity, and mean temperature were higher in 2020/2021, which could explain the spread of symptoms and increased disease severity for TS in comparison to 2018/2019 (Figure 2, Figure S2). The *Ptr*–wheat interaction follows an inverse gene-for-gene model, where susceptibility results from the unique interaction between Necrotrophic effectors (NEs) and specific receptors in the host, while the lack of NE recognition by the host leads to resistance. Hence, the best strategy for breeding against tan spot disease would couple the elimination of susceptibility genes with the introgression of genes that confer resistance at all growth stages to multiple races, as illustrated in previous studies on tan spot resistance that support the involvement of several NE–host interactions that may depend on the host growth stage [34,53,54,55,57,58]. The inheritance of resistance to tan spot is known to be both qualitative and quantitative [34,35,59,60,61,62,63,64,65] and to date, nine major *Tsr* genes (*Tsrl-Tsr7*, *TsrHar* and *TsrAri*) have been identified [66,67,68,69,70,71,72]. As the accessions in this study were tested in a region where multiple races were identified, a quantitative form of resistance to *Ptr* that is non-race specific could be identified, particularly in the accessions that exhibited R-MR reactions [34,65]. Moreover, accessions with a common name but different PI/Cltr numbers (USDA reference identifier) that may be similar or different were compared in this study. The geographical origin of accessions was found to have a significant effect on adult disease scores during the two years of trials. Populations from Portugal had the highest level of resistance (R-MR) at the adult stage over the years of trials, compared to populations from Algeria, France, and Italy. In fact, all populations from Portugal maintained a good level of resistance (above 50% R-MR). Populations such as ‘Oued Zenati’ from Algeria and ‘Gerardo’ from Italy showed low levels of resistance across the two-year trials. Levels of resistance/susceptibility of populations with the same common name but originating from different regions were also compared in this study. The ‘Raspinegro’ population was R-MR independent of the testing season or origin of accessions, suggesting that these accessions probably have a common origin or that seed exchange occurred between Portugal and Spain. Other populations, such as ‘Bidi’ and ‘Russo’, displayed a variable level of resistance and susceptibility with regard to the country of origin of the accessions, indicating that these accessions may be different despite of their common name. Therefore, genotyping of these accessions could help in identifying their origin as well as their genetic diversity. The origin of the accessions along with the phenotypic and genotypic diversity of *Ptr* populations in the NWR of Tunisia [25], where this study was conducted, may have an effect on the disease response of accessions, similar to the study of Dinglasan et al. [55], where they hypothesized that the prevalence of diverse *Ptr* populations was a main factor influencing wheat selection pressure and that accessions with all-stage resistance genes (ASR) and APR genes had different geographical origins. In addition, a high diversity was observed when comparing the frequency of resistance (R-MR) of all accessions based on their level of improvement at both growth stages. Genetic material and Landraces were the most resistant, while cultivars were the least resistant. The analysis of variance showed a significant effect of the level of improvement of accessions on adult resistance. These finding further highlight the importance of exploitation of landraces in breeding programs for TS resistance and the introgression for novel resistance genes into modern cultivars.

In addition, in this study, PH was significantly negatively correlated to adult-stage resistance, indicating that the shorter plants are the more susceptible to TS. This could also indicate an escape effect, as illustrated in the study of Muqaddasi et al. [73], as taller plants have more space between nodes, thus making the spread of conidia from lower leaves to upper leaves difficult. This suggests that selection for increased height would help in contributing to escape from TS. Many studies delt with correlation between wheat resistance to TS and PH, where conflicting results were found; while some studies concluded that there was no significant effect of PH on TS development [14,51,74,75], other studies concluded that plant height may affect TS severity [73,76]. Many factors could have contributed to these different outcomes, including the use of genetically different germplasms, the size of the panel, the conduction of these experiments in different environments, environmental or epidemiological factors, and the variable *Ptr* populations used in the conduction of these studies. The cluster analysis based on rAUDPC, and PH over the two cropping seasons, revealed three clusters. Cluster 1 included 96.5% and 84.7% of R-MR accessions in 2019 and 2021, respectively, while MS-S accessions were mostly included in Cluster 2 and MR-MS were mostly included in Cluster 3. These results suggest that accessions from Cluster 1 are most suitable for use in breeding programs for tan spot resistance. The second cluster (Figure 7b) included 59 accessions with R or S reaction to TS over the two years of trials, and allowed for the classification of wheat accessions into three different clusters. In total, 93.5% of resistant accessions over the two years of trials were included in Cluster 3 separately from susceptible accessions that were grouped in Clusters 1 and 2, suggesting that accessions of Cluster 3 are most suitable for use in breeding programs for TS resistance. In this study, although days of heading (DH) was not investigated, it could be a trait that may have an effect on tan spot severity, particularly given that several studies tested the effect of DH on tan spot development and reported contradictory conclusions. While some concluded that DH was no correlated to TS resistance/susceptibility [74,75,76], others suggested that DH may have an effect on TS severity [14,73]. Hence, follow-up studies could shed further light on the contribution of DH in disease development.

When comparing the resistance of 538 accessions to both STB and TS diseases, the resistance levels of STB between 2016/2017 and 2018/2019 were not variable; meanwhile, the number of R accessions to TS decreased in favor of MR-MS accessions. This can be partially attributed to the homothallic nature of *Ptr* compared to the heterothallic nature of *Z. tritici*. In heterothallic fungi such as *Z. tritici*, sexual reproduction occurs uniquely after the mating of two strains of opposite mating types, whereas in homothallic fungi, such as *Ptr*, sexual reproduction can occur even within the same clone, which increases its spread [77,78,79]. Thus, the availability of compatible mating type for *Z. tritici* may limit its sexual reproduction and hinder overwintering survival [79,80]. Furthermore, increased disease prevalence in heterothallic fungi was shown to increase the probability of contact between the two mating types [81,82]. Meanwhile in homothallic fungi, this mechanism is absent, making critical the environmental signals triggering the production of the resting structures [79]. Indeed, in Tunisia, the occurrence of TS infection in wheat fields before STB [83], coupled with the environmental conditions (Figure S2) that are conducive to TS development, along with the homothallic nature of *Ptr*, could lead to a fast spread of TS and an increase in inoculum density [79,82]. Inoculum density decreases the latency period; since *Z. tritici* has a longer latent period (10–14 days) compared to *Ptr* (5–7 days) [79,82], this allows for faster establishment of TS. Therefore, the high genetic diversity and fast spread of *Ptr* could lead to resistance breakdown faster than that of *Z. tritici.* Climatic conditions were also more favorable for TS disease than STB within the studied years. In this study, almost half of the 538 accessions maintained an R-MR reaction for both diseases; these accessions can be exploited simultaneously for STB and TS resistance breeding. The overall results of this study suggest that the accessions with R-MR reaction types to TS and STB diseases can be used to identify possible novel APR genes/QTLs that confer resistance to the two main wheat-threatening diseases in the Mediterranean region, and Tunisia in particular.

## 5. Conclusions

The results show that the USDA collection of Mediterranean durum wheat accessions tested in this study could provide a good and diverse source of resistance to both tan spot and Septoria tritici blotch diseases. The genotype was found to be highly significant at both the seedling and adult stages, emphasizing the high genetic variability of the tested accessions. PH was found to have a significant negative effect on adult-stage resistance, suggesting that selecting for taller genotypes can increase TS resistance. When comparing reaction types among and between countries, high diversity was observed with regard to TS resistance. Some accessions with the same name or different origins were found to have different reactions to TS, suggesting that these accessions may in fact be different genetically. In total, 69% of accessions showed a similar resistance level at both seedling and adult stages, indicating that these accessions may harbor potential novel QTLs/genes. Nearly half of the accessions were found to be resistant to both TS and STB over two years, suggesting that these accessions could harbor effective multi-resistance genes. Hence, genotyping of landrace accessions could reveal important genetic diversity that could be linked to phenotypic traits. Investigating the genetic diversity of landrace accessions, using those that exhibited R-MR reactions, could eventually lead to discovering ‘new’ allelic variation for TS and STB to be effectively exploited in breeding programs.

## Figures and Tables

**Table 1:** Adult-stage reaction types of the Mediterranean wheat accessions against *Pyrenophora tritici-repentis* under field conditions.

Reaction Types ^1^	rAUDPC Range	Symptom’s Description
R	<0.5	small apparent infection at low leaves
MR	0.5–0.7	Midheight infection and apparent lesions not well developed
MS	0.7–0.9	Well-developed lesions up to F^−1^ *
S	>0.9	Well-developed lesions up to flag leaf

^1^ R: resistant, MR: moderately resistant, MS: moderately susceptible, S: susceptible. * F^−1^ corresponds to the leaf below the flag leaf.

**Table 2:** Pearson’s correlation coefficient for the disease parameters and plant height among wheat genotypes across seasons (2018–2019 and 2020–2021).

Variables	rAUDPC 2019	PH 2021
Seedling 2019	*r* = 0.289 *p* =4.685 × 10^−11^ ***	-
PH 2019	*r* = −0.086 *p* = 0.045 *	*r* = 0.605 *p* < 2.2 × 10^−16^ ***
rAUDPC 2021	*r* = 0.531 *p* < 2.2 × 10^−16^ ***	*r* = −0.347 *p* < 2.2 × 10^−16^ ***

rAUDPC = relative area under the disease progress curve; PH = plant height. Signifiance codes: *** 0.001; * 0.05

**Table 3:** Analysis of variance (ANOVA) of the relative area under the disease progress curve on the Mediterranean wheat accessions during two cropping seasons (2018–2019 and 2020–2021).

Physiological Stage	Source of Variation	df ^1^	Sum of Squares	Mean of Squares	F Value	Pr (>F)
**Adult**	Year	1	7.8	7.755	9.064	0.00267 **
	residuals	1096	937.7	0.856		
	Genotype	548	658.0	1.2007	2.293	<2 × 10^−16^ ***
	residuals	549	287.5	0.5237		
	Genotype × Year	547	8.722	0.01594	6.532	0.3042
	residuals	1	0.002	0.00244		
	Origin	4	150.0	37.49	51.51	<2 × 10^−16^ ***
	Residuals	1093	795.5	0.73		
	Level of improvement	4	60.9	15.223	18.9	5.02 × 10^−15^ ***
	Residuals	1071	862.8	0.806		
	PH	27	3.99	0.14796	4.98	4.19 × 10^−15^ ***
	Residuals	1070	31.79	0.02971		
	Seedling	3	41.4	13.794	14.21	6.7 × 10^−9^ ***
	Residuals	503	488.4	0.971		

^1^ df: Degrees of freedom; Signifiance codes: *** 0.001; ** 0.01.

**Table 4:** Frequency of resistance (% R-MR) at adult and seedling stages in 2018–2019 and 2020–2021 cropping seasons of 549 accessions used in this study according to their level of improvement.

Level of Improvement	% R-MR Seedling 2019	% R-MR Adult 2019	% R-MR Adult 2021	% R-MR Adult 2019 and 2021
Breeding material	50	56	52	39
Cultivar	30	32	25	18
Genetic material	69	92	92	85
Landrace	61	65	65	49
Unknown improvement status	73	67	51	37

R: resistant; MR: moderately resistant.

## Data Availability

The data presented in this study are available within the article.

## Supplementary Materials

The following supporting information can be downloaded at: https://www.mdpi.com/article/10.3390/genes13020336/s1, Figure S1. Origin of 549 accessions tested over 2-year trials (2018–2019 and 2020–2021) at the CRP-Wheat Septoria Platform, Experimental station of Kodia (Bou Salem, Tunisia), Figure S2. Monthly precipitations and relative humidity (HR) (distributions (bar)), and temperature variation (minimum, mean, and maximum) (line) during the two cropping seasons 2018–2019 and 2020–2021 at Kodia experimental station, Figure S3. Morphological characteristics of *Pyrenophora tritici-repentis* (a) and (b) Conidia (red arrow) and conidiophores (blue arrow) developed on the surface of infected leaf, (c) *Ptr* conidia, (d) Pseudothecia formed on straw, (e) Erupted pseudothecium with asci and ascospores, (f) Mature asci and ascospores, Table S1. Data on disease evaluation based on seedling and adult scores (AUDPC and rAUDPC) under field conditions as well as Plant height of the Mediterranean collection (549 accessions) over two seasons, Table S2. Ranking of mean Area Under Disease Progress curve (AUDPC) of checks (INRAT 100, Karim, Salim and Nasr) evaluated in field during two cropping seasons against tan spot.
