# Supplementary material for: Characterization of Mediterranean Durum Wheat for Resistance to Pyrenophora tritici-repentis
Source: Genes (Basel). 2022 Feb 11;13(2):336. doi: 10.3390/genes13020336 (PMC8872616; doi:10.3390/genes13020336)
Supplement: Supplementary file 1 [file genes-13-00336-s001.zip › Supplementary Figures.pdf]

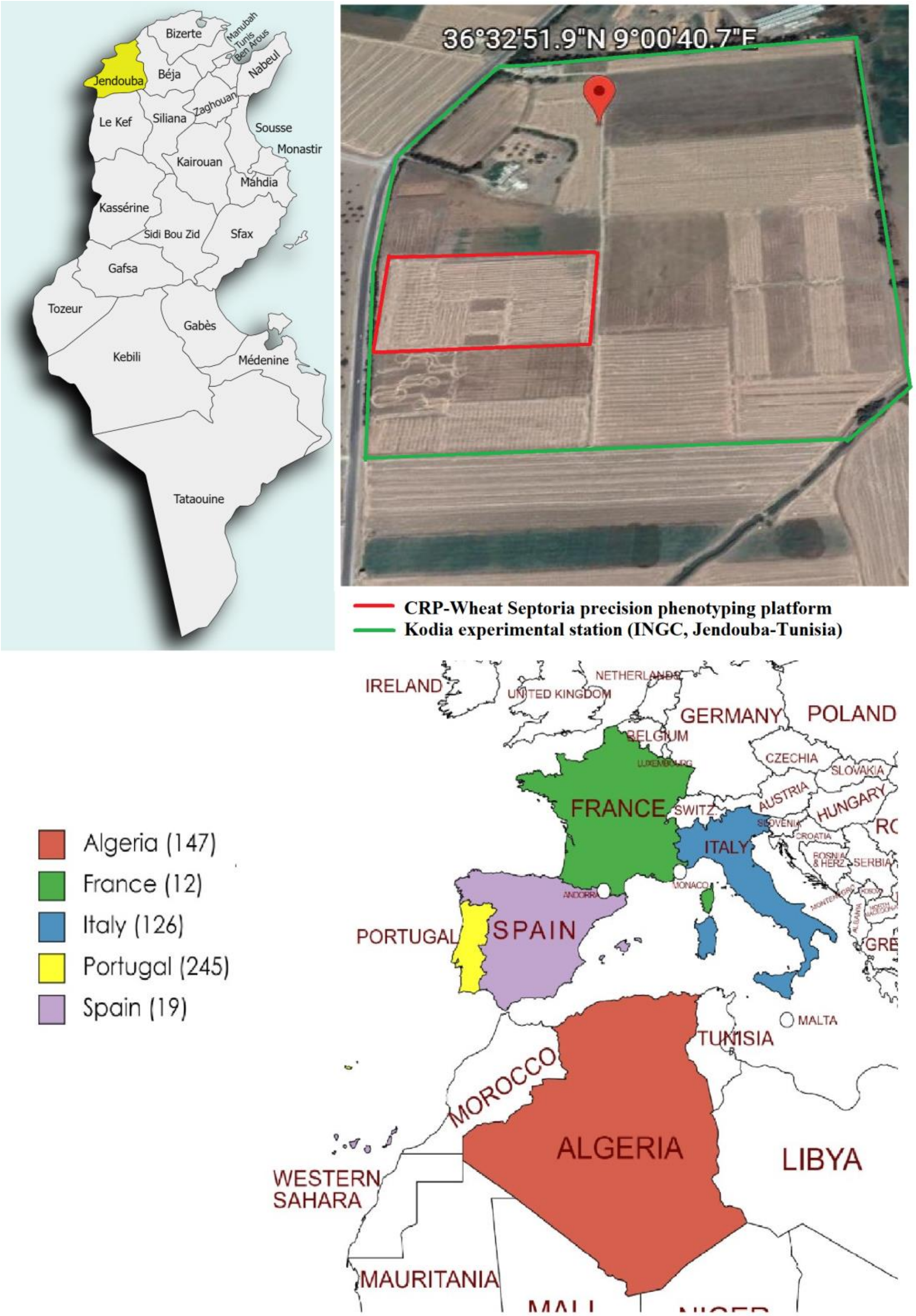

Figure S1. Origin of 549 accessions tested over 2-year trials (2018-2019 and 2020-2021) at the CRP-Wheat Septoria Platform, Experimental station of Kodia (Bou Salem, Tunisia)

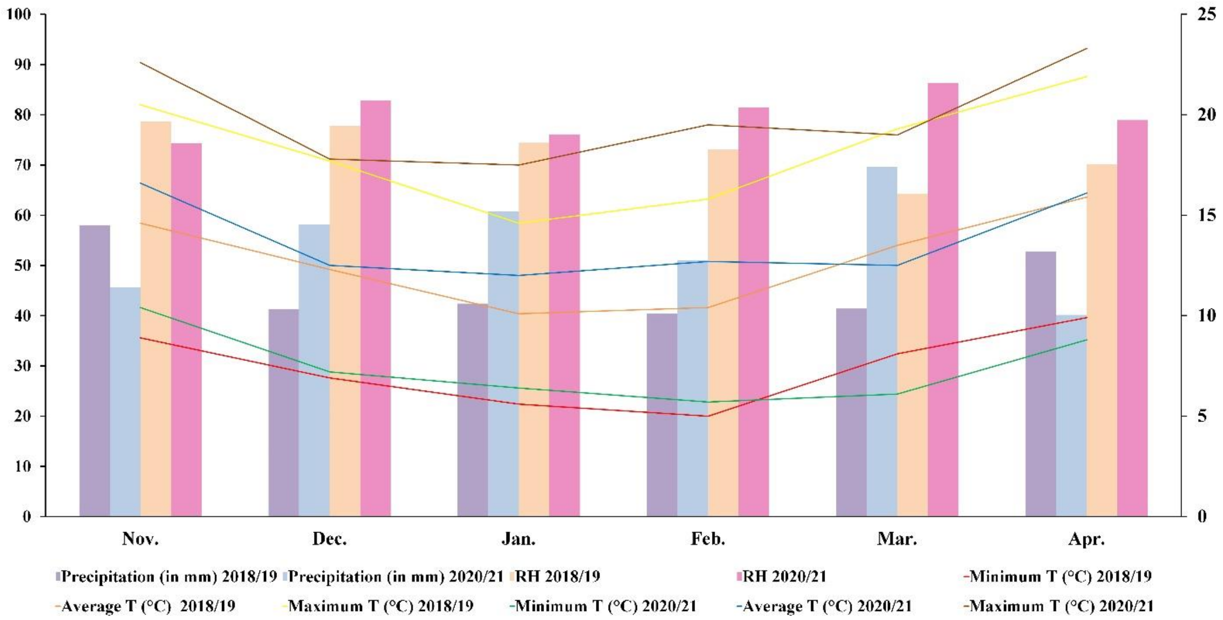

Figure S2. Monthly precipitations and relative humidity (RH) (distributions (bar)), and temperature variation (minimum, mean, and maximum) (line) during the two cropping seasons 2018-2019 and 2020-2021 at kodia experimental station.

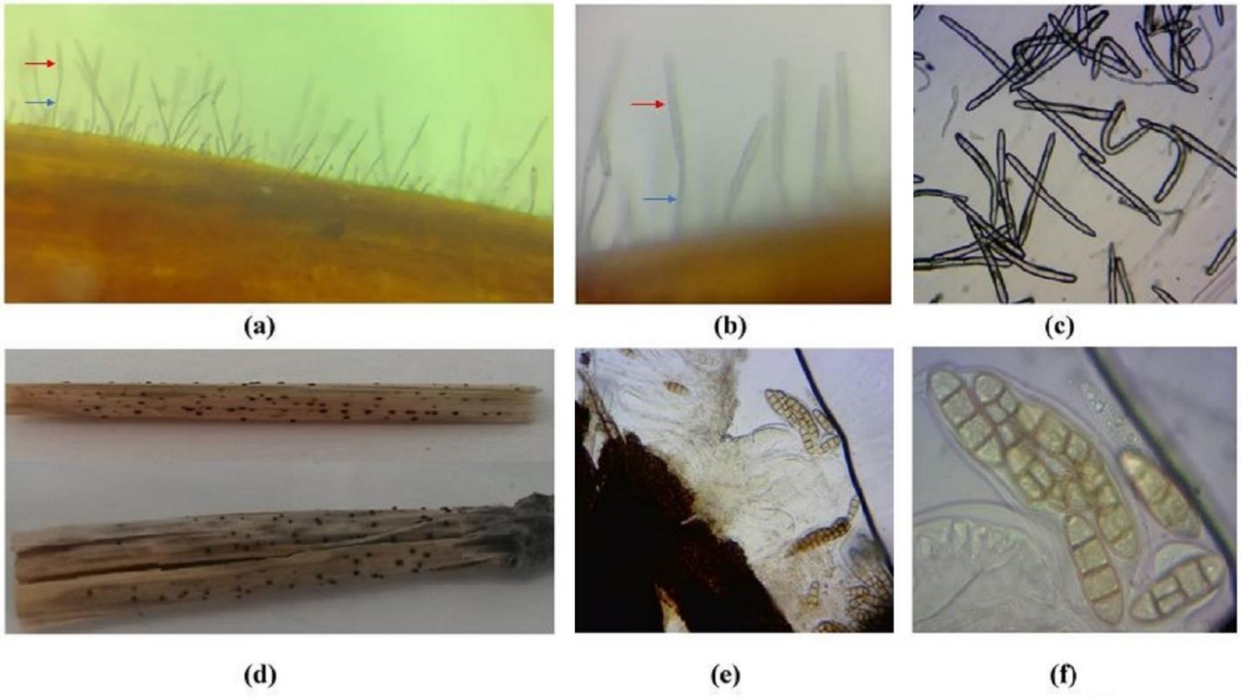

Figure S3. Morphological characteristics of *Pyrenophora tritici-repentis* (a) and (b) Conidia (red arrow) and conidiophores (blue arrow) developed on the surface of infected leaf, (c) *Ptr* conidia, (d) Pseudothecia formed on straw, (e) Erupted pseudothecium with asci and ascospores, (f) Mature asci and ascospores
