# Supplementary figures and images for: Characterization of Mediterranean Durum Wheat for Resistance to Pyrenophora tritici-repentis
Source: Genes (Basel). 2022 Feb 11;13(2):336. doi: 10.3390/genes13020336 (PMC8872616; doi:10.3390/genes13020336)

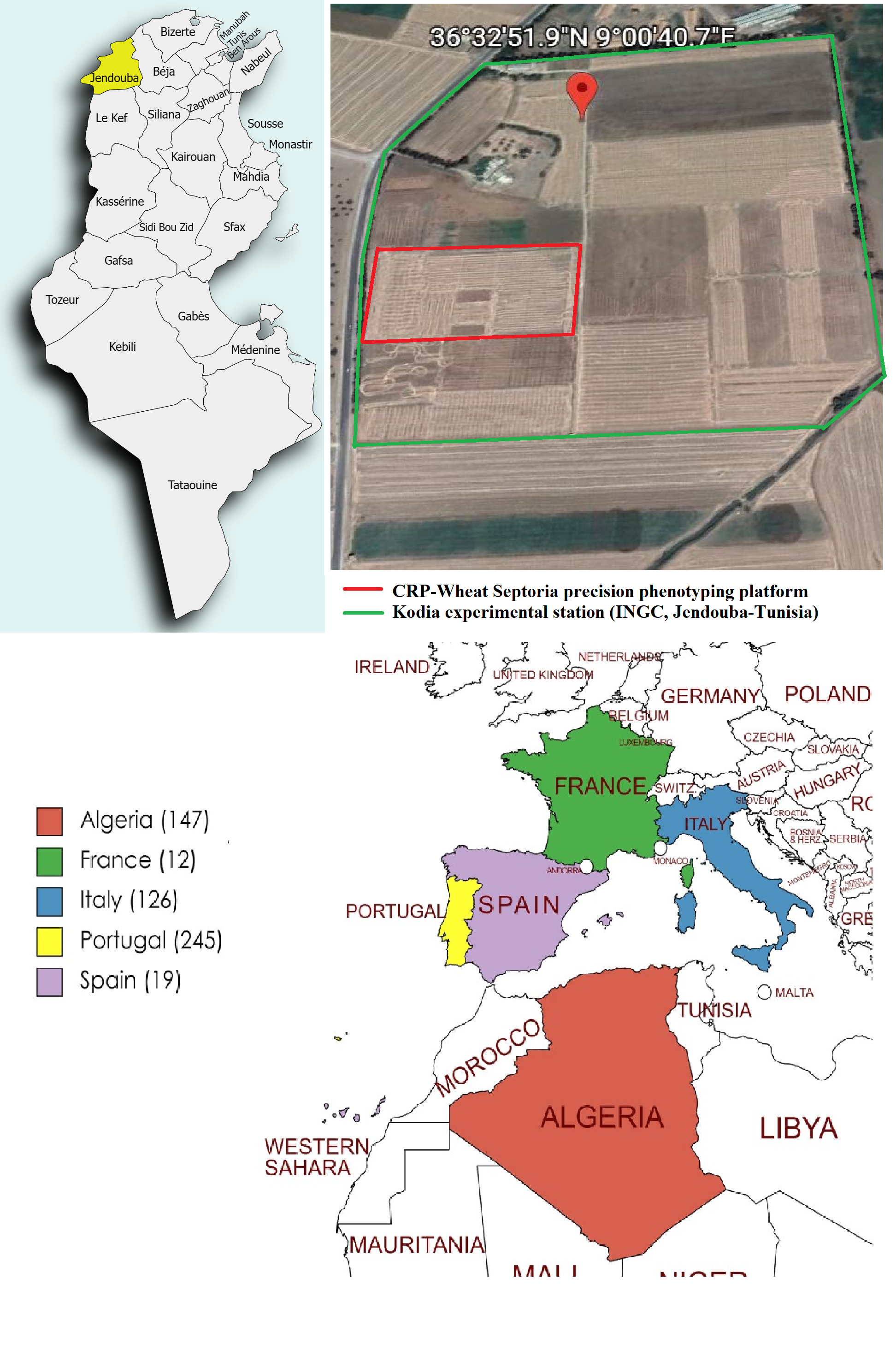

Supplement: Supplementary file 1 [file genes-13-00336-s001.zip › Figure S1.png]

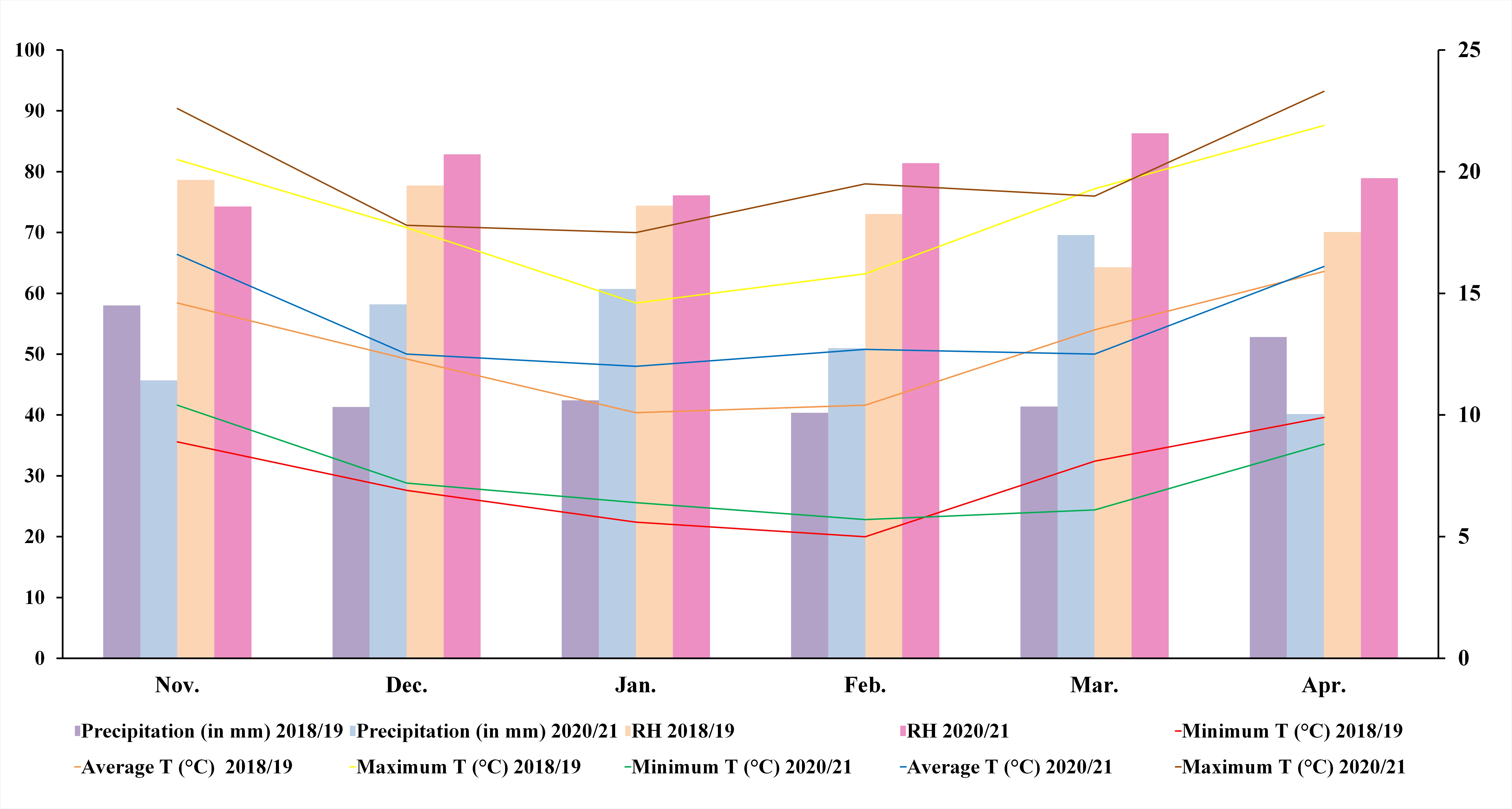

Supplement: Supplementary file 1 [file genes-13-00336-s001.zip › Figure S2.jpg]

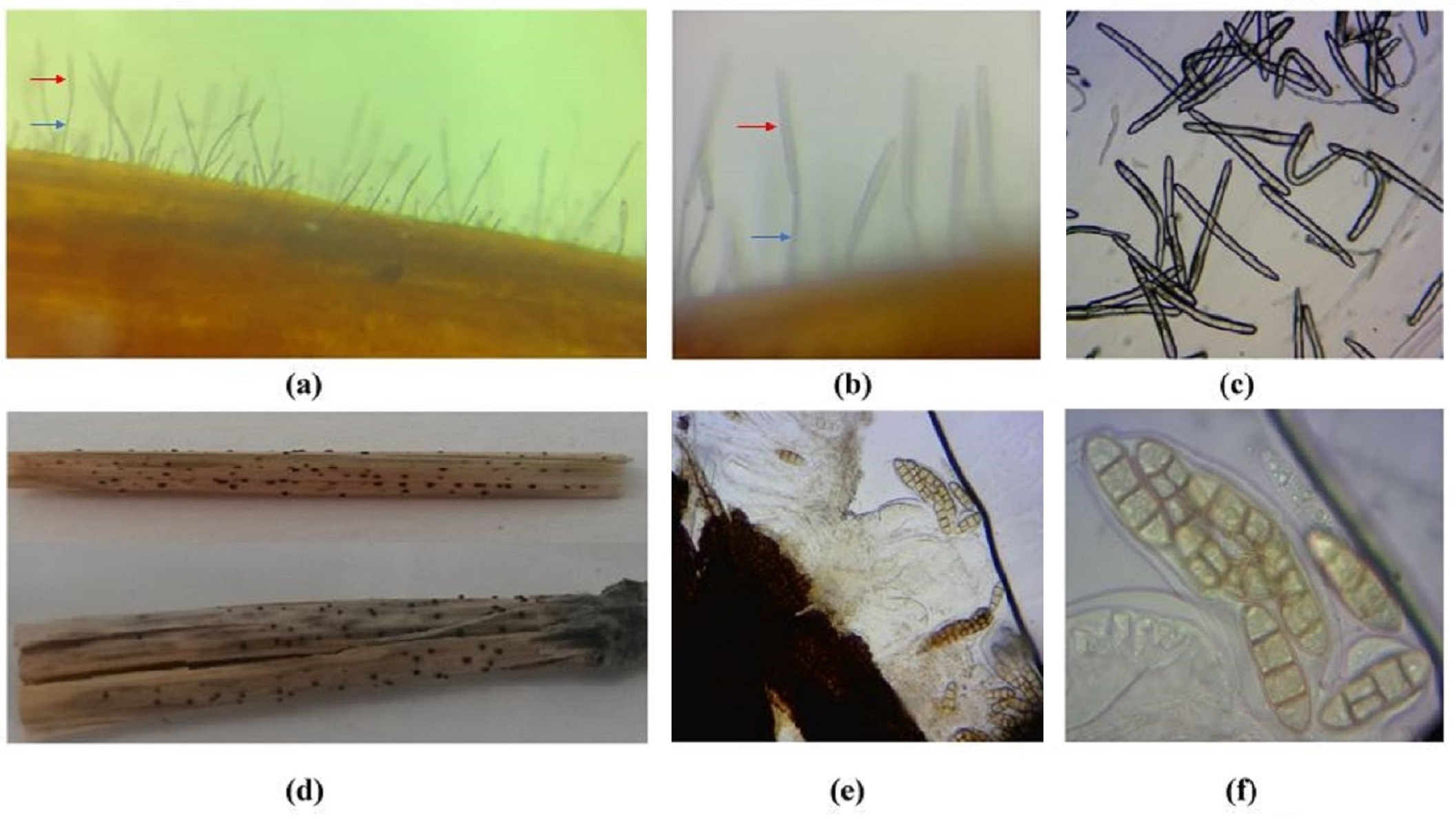

Supplement: Supplementary file 1 [file genes-13-00336-s001.zip › Figure S3.JPG]
